# Prognostic significance of CDC20 expression in malignancy patients: A meta-analysis

**DOI:** 10.3389/fonc.2022.1017864

**Published:** 2022-11-21

**Authors:** Feng Xian, Xuegang Yang, Guohui Xu

**Affiliations:** ^1^ School of Medicine, University of Electronic Science and Technology of China, Chengdu, China; ^2^ Oncology Department, Nanchong Central Hospital, The Second Clinical Institute of North Sichuan Medical College, Nanchong, China; ^3^ Department of Interventional Radiology, Sichuan Cancer Hospital and Institute, Sichuan Cancer Center, School of Medicine, University of Electronic Science and Technology of China, Chengdu, China

**Keywords:** CDC20, prognosis, malignancy, meta-analysis, overall survival

## Abstract

**Background:**

Cell Division Cycle Protein 20(CDC20) is reported to promote cancer initiation, progression and drug resistance in many preclinical models and is demonstrated in human cancer tissues. However, the correlation between CDC20 and cancer patients’ prognosis has not yet been systematically evaluated. Therefore, this present meta-analysis was performed to determine the prognostic value of CDC20 expression in various malignancy tumors.

**Methods:**

A thorough database search was performed in EMBASE, PubMed, Cochrane Library and Web of Science from inception to May 2022. Stata14.0 Software was used for the statistical analysis. The pooled hazard ratios(HRs) and their 95% confidence intervals (95% CIs) were used to analysis of overall survival (OS), recurrence-free survival (RFS), distant-metastasis free survival (DMFS). Qualities of the included literature were assessed by JBI Critical appraisal checklist. Egger’s test was used to assess publication bias in the included studies.

**Results:**

Ten articles were selected, and 2342 cancer patients were enrolled. The cancer types include breast, colorectal, lung, gastric, oral, prostate, urothelial bladder cancer, and hepatocellular carcinoma. The result showed strong significant associations between high expression of CDC20 and endpoints: OS (HR 2.52, 95%CI 2.13-2.99; HR 2.05, 95% CI 1.50-2.82, respectively) in the multivariate analysis and in the univariate analysis. Also, high expression of CDC20 was significantly connected with poor RFS (HR 2.08, 95%CI 1.46-2.98) and poor DMFS (HR 4.49, 95%CI 1.57-12.85). The subgroup analysis was also performed, which revealed that CDC20 upregulated expression was related to poor OS in non-small cell lung cancer (HR 2.40, 95% CI 1.91-3.02).

**Conclusions:**

This meta-analysis demonstrated that highly expressing CDC20 was associated with poor survival in human malignancy tumors. CDC20 may be a valuable prognostic predictive biomarker and a potential therapeutic target in various cancer parents.

## Introduction

Cancer is a common cause of morbidity and mortality throughout the world (1). Worldwide, an estimated 19.3 million new cancer cases and almost 10.0 million cancer deaths occurred in 2020 (2). In spite of desperate development of new remedies in recent years, the prognosis of cancer remains bleak (3). Therefore, detection of new biomarkers related to the progression of cancer is essential for improving clinical outcomes.

It is known that CDC20 consists of 499 amino acids with WD40 repeats at its C-terminus for protein binding, serving as the substrate recognizing subunit of Anaphase Promoting Complex (APC) (4). CDC20 has been found to play critical roles in regulating timely cell cycle progression in both the G2 and M phases (5). Multiple studies from various groups have demonstrated that CDC20 targets several key substrates including Securin, Cyclin B1, Cyclin A, Nek2A, p21 and Mcl-1 for degradation to govern cell cycle progression (6–11). Moreover, some studies show that p53, Mad2, RASSF1A and APC15 could inhibits tumors cell growth through regulation of CDC20 (12–15). CDC20 have been proven to have kinds of functions, including regulation of cell cycle,and regulation of apoptosis. Mounting evidence has revealed that CDC20 plays an oncogenic role in human tumorigenesis, and increased CDC20 expression is associated with clinical progression in human cancers, such as poor differentiation and poor recurrence-free survival rates (16, 17). However, it remains unclear whether CDC20 is associated with a worse outcome across solid cancer patients. These conflicting results may be due to the small sample size among individual studies and limitation of current technology.

Therefore, we present a meta-analysis evaluating the prognostic value of CDC20 over-expression in solid tumor. The purpose of this study was to estimate the correlation of CDC20 over-expression with survival in solid tumors, thereby shed more light on the development of CDC20 targeted therapy and prognostic prediction.

## Materials and methods

### Search strategy

This study followed the Preferred Reporting Items for Systematics Reviews and Meta-Analysis (PRISMA) (18). We utilized a systematic search based on the PubMed, Embase, Web of Science and Cochrane Library from the establishment of data to May 2022. The search terms included (“cell division cycle protein 20” or “CDC20”) and (“neoplasms” or “tumors” or “cancers” or “carcinoma” or “malignancies”) and (“prognosis” or “survival”). All potentially eligible studies were retrieved, and their bibliographies were carefully scanned to identify other eligible studies and extra studies were identified by a hand search of the references cited in the original studies. When multiple studies of the same patient population were identified, we included the published report with the largest sample size. The above search process was done by two reviewers independently.

### Inclusion criteria

To be eligible for inclusion in this meta-analysis and data extraction, studies had to: (1) Patients were pathologically diagnosed with any type of malignancy. (2) The expression levels of CDC20 were identified in tissues samples. (3) Patients were classified into negative and positive expression or low and high expression group in line with the CDC20 of expression levels, the connection between expressing level of CDC20 and survival results was examined. (4) HR and 95% confidence intervals (CI) for survival times were computed by included articles which can provide enough data or survival curves. (5) Officially published and English-written literatures.

### Exclusion criteria

Exclusion criteria were: (1) Duplicated articles; (2) literatures published as letters, editorials, abstracts, reviews, case reports and export opinions; (3) experiments performed *in vitro* or *in vivo*, but not based on patients; (4) insufficient data about survival analysis; (5) the follow-up duration was shorter than 3 years.

### Data extraction

Two reviewers extracted related data from the articles independently and came to an agreement on the following items. Original data of elementary demographic characteristics (authors of article; year of publication; detection method; age; region; CDC20 level; the category of carcinoma; follow-up duration; endpoints; the Joanna Briggs Institute (JBI) score) were exhaustively extracted from included literature involving Kaplan-Meier curves, test words and tables. In term of endpoints, OS, RFS and DMFS were considered as terminal events. For some articles, HR can be directly obtained; for the studies in which survival data are presented only with K-M curves, Tierney’s method was employ was used to calculate the HR and 95% CI (19).

### Methodological assessment

Two investigators individually assessed qualities of all enrolled studies by utilizing the JBI (20). The JBI Critical appraisal checklist for observational cohort study included 11 items. We regarded the included studies with at least 15 score as high-quality in methodology. And if the score were less than 15, those articles were considered as low-quality studies.

### Statistical analysis

Our quantitative calculation was conducted based on Stata Software 14.0. We applied pooled HRs (high/low) along with its related 95% CIs to evaluate the association between the prognostic value and the expression levels of CDC20 in different malignancies. By utilizing Cochran’s Q and I^2^ statistics, the heterogeneity of enrolled literatures can be evaluated precisely. Additionally, Chi square-based Chochran Q test and I^2^ test were calculated to determine the heterogeneity among these articles (21). Heterogeneity was considered insignificant when p>0.10 or I^2^ < 50%, and then fixed-effects was employed to pool the HRs and 95% CIs; Otherwise, a fixed-effects model was used.

In order to explore the source of heterogeneity, we also performed subgroup analysis and meta-regression. Furthermore, sensitivity analysis was implemented to confirm the steadiness of collected results. Finally, we assessed publication bias by means of utilizing Egger’s test. What’s more, if the p-value is no more than 0.05, the results above all can be regarded as statistical significance.

## Results

### Characteristics of studies

**Figure 1:**
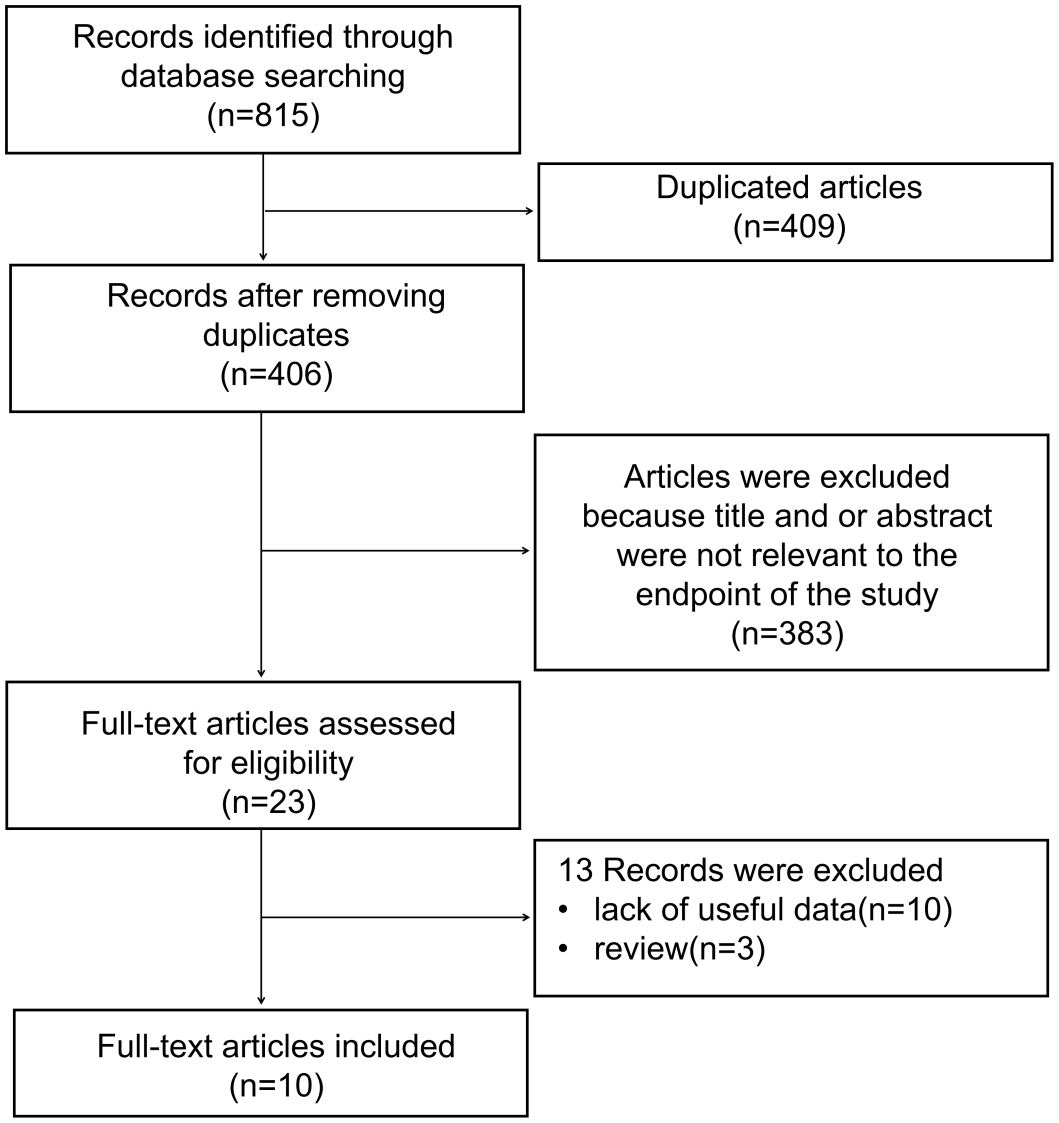
The flow chart of the selection process in our meta-analysis.

Eventually, ten studies (22–31) involving a total of 2342 patients were used for the meta-analysis (**Figure 1**). The included studies are summarized in **Table 1**. Two studies evaluated lung cancer, two studies evaluated breast cancer, and one each evaluated colorectal cancer, hepatocellular carcinoma, gastric cancer, oral cancer, urothelial bladder cancer, prostate cancer. The studies were performed in six countries (People’s Republic of China, Finland, United King, Gandra, Korea, Japan) and published prior to May 2022.

**Table 1:** Characteristics of the included studies.

References	Year	Country	Cancer type	Age (years)	Case	Method	Increased CDC20(%)	Follow-up(months)	Endpoints	JBI
Karra et al. (22)	2014	Finland	breast cancer	61	445	IHC	165(37.1%)	120	OS(M)	20
Alfarsi et al. (23)	2019	UK	breast cancer	–	347	IHC	–	60	RFS、DMFS	20
Wu et al. (24)	2013	China	colorectal cancer	85/159(≤50/>50)	244	IHC	114(46.7%)	91	OS(M)	20
Shi et al. (25)	2017	China	Lung cancer	26/78((<60/≥60)	104	qRT-PCR	107(99.1%)	240	OS(M)	22
Zhang et al. (26)	2021	China	hepatocellular carcinoma	69/70(<60/≥60)	139	qRT-PCR	74(53.2%)	60	OS(M)、OS(U)	20
Ding et al. (27)	2014	China	gastric cancer	47/84(<60/≥60)	131	IHC	68(51.9%)	60	OS(M)、OS(U)	20
Moura et al. (28)	2013	Gandra	Oral cancer	32/33(<62/≥62)	65	IHC	37(56.9%)	120	OS(M)	20
Choi et al. (29)	2013	Korea	urothelial bladder cancer	68	339	IHC	200(59%)	37	RFS、DMFS	22
Mao et al. (30)	2016	China	prostate cancer	65.2	166	IHC	40(24.1%)	90	OS(M)、OS(U)	22
Kato et al. (31)	2012	Japan	Lung cancer	63.5	362	IHC	71(19.6%)	60	OS(M)	20

-, not provided; IHC, immunohistochemistry; qRT-PCR, quantitative real-time polymerase chain reaction; OS(M), overall survival (multivariate analysis); OS(U), overall survival(nivariate analysis); RFS, recurrence-free survival; DMFS, distant-metastasis free survival; JBI, the Joanna Briggs Institute.

### Relationship between CDC20 expression level and OS of malignancy patients

**Figure 2:**
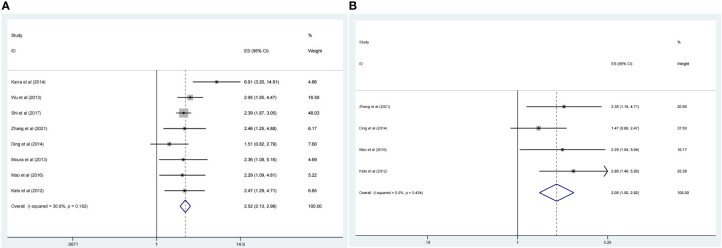
Forest plot of meta-analysis of the relationship between CDC20 expression and OS of malignancy patients. **(A)** HR of OS in the multivariate analysis, **(B)** HR of OS in the univariate analysis.

**Figure 3:**
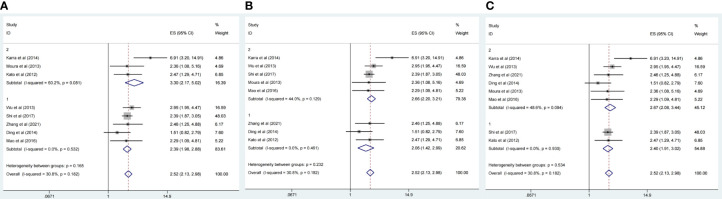
Forest plot of meta-analysis of the relationship between CDC20expresison and OS of malignancy patients as different factors. **(A)** Subgroup analysis stratified by type of cancers [lung cancer (group 1) and other cancers (group 2)]. **(B)** Subgroup analysis stratified by follow-up time [over 60 months (group1) or less than 60 months (group2)]. **(C)** Subgroup analysis stratified by country [China (group1) or other countries (group2)].

There were eight studies that reported OS data with multivariate analysis. The relevant results showed that CDC20 overexpression in human tumor tissues was associated with a decrease in survival among malignancy patients (HR 2.52, 95% CI 2.13-2.99, p <0.001) in the multivariate analysis and (HR 2.05, 95% CI 1.50-2.82, p <0.001) in the univariate analysis (**Figures 2A, B**). There was slight heterogeneity among the eight studies mentioned (*P* =0.18, *I* ^2^ = 30.8%). Subgroup analyses were conducted as different factors including type of cancer (lung cancer or other cancers), follow-up duration (over 60 or less than 60 months), country (China or other country), and the pooled HRs for OS were shown in **Figures 3A–C**. The results of subgroup analysis showed that the high expression of CDC20 was associated with poor OS of malignancy patients (**Table 2**).

**Table 2:** Subgroup analysis of pooled HRs for OS in cancer patients with abnormal expression level of CDC20.

Subgroup analysis	No. of studies	Pooled or Random	Meta regression (p value)	Heterogeneity	
				I^2^(%)	P value
Type of cancer			<0.001		
Lung cancer	2	2.40 [1.91-3.02]		0.0	0.930
Non-lung cancer	6	2.67 [2.08-3.44]		48.6	0.084
Follow-up time			<0.001		
≤ 60 months	3	2.06 [1.42-2.99]		0.0	0.461
> 60months	5	2.66 [2.20-3.21]		44.0	0.129
Coutry			<0.001		
China	5	2.39 [1.98-2.88]		0.0	0.532
Other countries except china	3	3.30 [2.17-5.02]		60.2	0.081

### Relationship between the expression of CDC20 and OS of lung cancer patients

**Figure 4:**
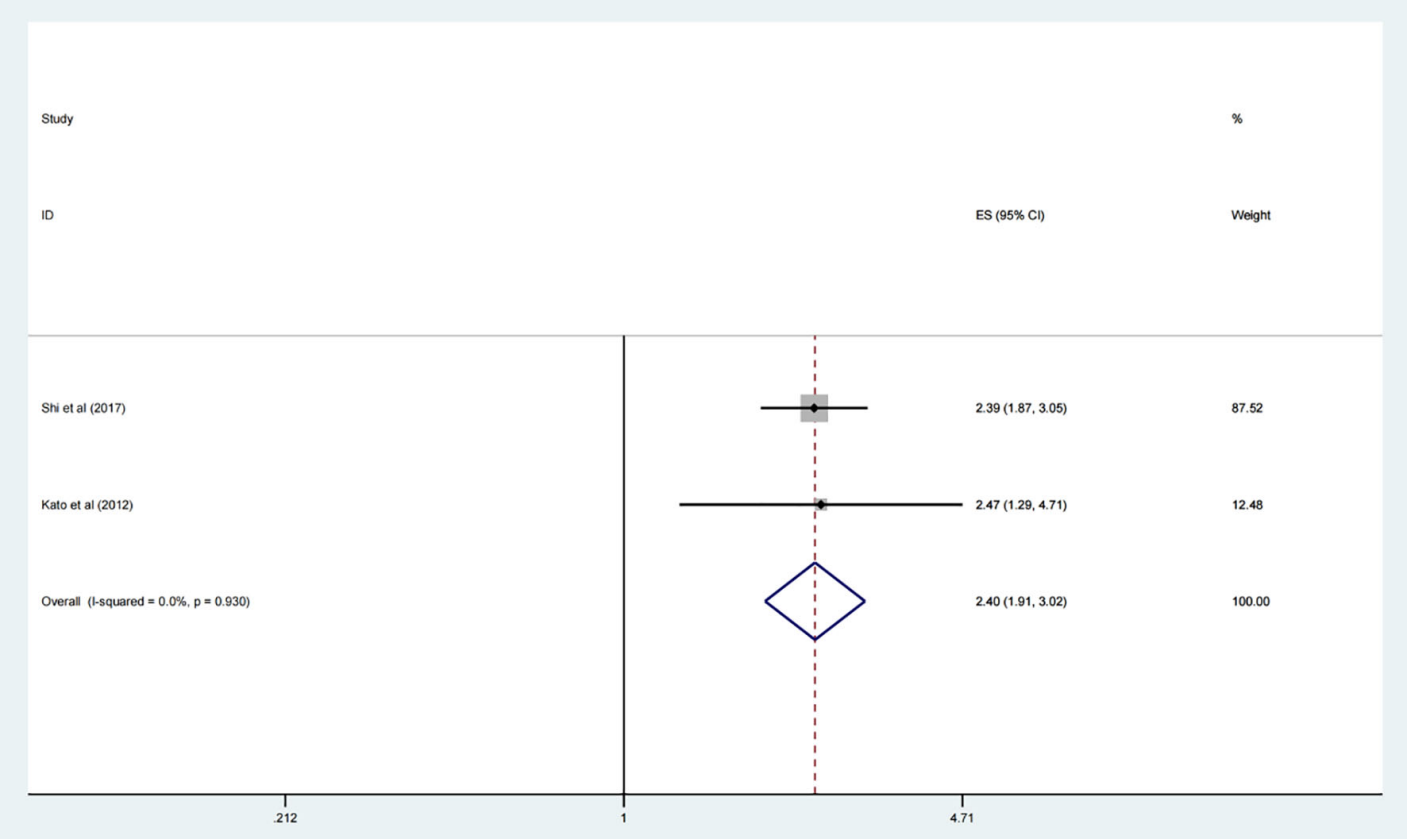
Forest plot of meta-analysis of the relationship between CDC20 expression and OS of lung cancer patients.

Additionally, the prognosis role of the expression levels of CDC20 in lung cancer was assessed systemically. The results suggested that elevated CDC20 level implicated an unfavorable OS in lung cancer (HR 2.40, 95% CI 1.91, 3.02, p <0.001) (**Figure 4**).

### Relationship between CDC20 expression level and RFS, DMFS of malignancy patients

**Figure 5:**
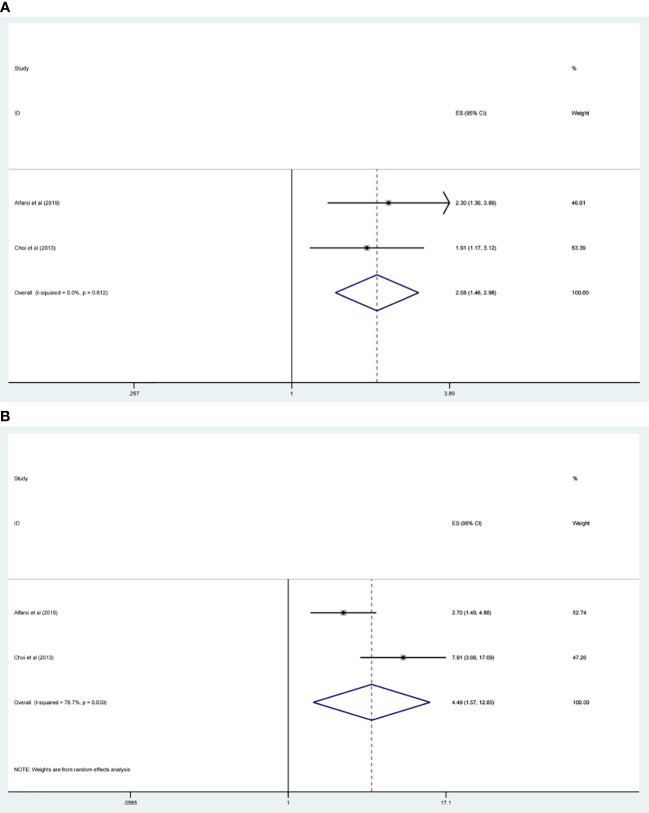
Forest plot of meta-analysis of the relationship between CDC20 expression and RFS **(A)** and DMFS **(B)** of malignancy patients.

Among the included studies, two research estimated the relevance between CDC20 expression level and RFS, DMFS, respectively. The results showed that CDC20 increasingly expression was significantly related with poor RFS (HR 2.08, 95% CI 1.46, 2.98, p <0.001) (**Figure 5A**) and DMFS (HR 4.49, 95% CI 1.57, 12.85, p <0.001) (**Figure 5B**). However, we did not perform subgroup analysis for other types of cancer because of the insufficient numbers of trials included.

### Sensitivity analysis

**Figure 6:**
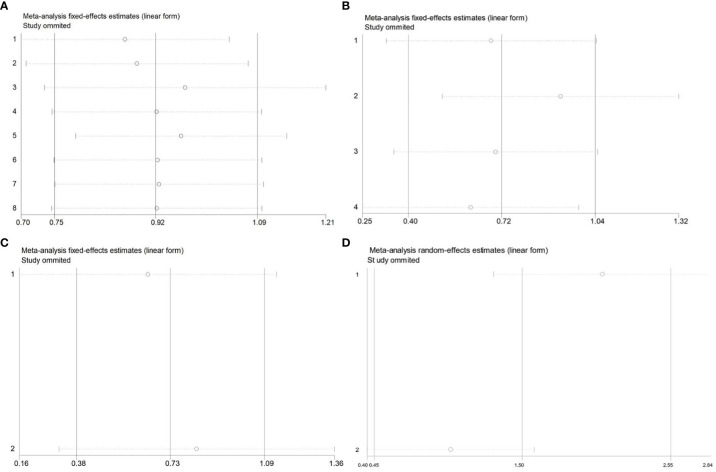
Sensitivity analysis of the meta-analysis. **(A)** OS of CDC20 expression levels in the multivariate analysis. **(B)** OS of CDC20 expression levels in the univariate analysis. **(C)** RFS of CDC20 expression levels. **(D)** DMFS of CDC20 expression levels.

In order to assess the impacts of single study on the total outcomes, sensitivity analysis was conducted. AS to OS, our result of sensitivity analysis revealed that all the outcomes could not influence consequences remarkably, which means that the outcomes of OS were stable. The list of pooled HRs and 95% CIs after excluding single study one by one indicated the robustness of our results (**Figures 6A, B**). Furthermore, the sensitivity analysis RFS (**Figure 6C**) and DMFS (**Figure 6D**) identified that each included study influenced outcomes greatly, which suggested that the results of RFS and DMFS were not stable because of the limited number of studies included in each analysis. Thus, more related studies were needed to explore the effects of CDC20 on RFS and DMFS in human malignancy.

### Publication bias

**Figure 7:**
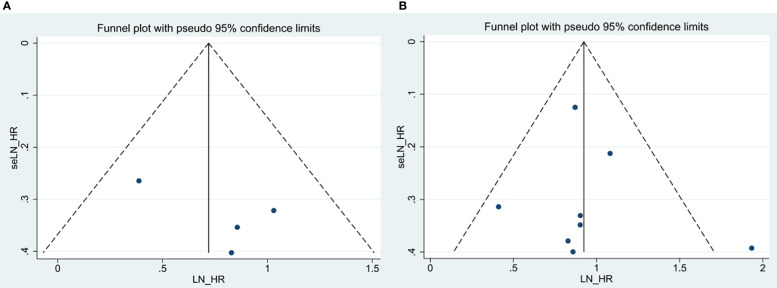
Egger’s funnel plots for the studies involved in the meta-analysis. **(A)** OS in the multivariate analysis. **(B)** OS in the univariate analysis.

By Egger’s test, we systemically assessed publication bias of all above included studies. The result of Egger’s test (p =0.664) (**Figures 7A, B**) about OS revealed that there existed no significant publication bias among enrolled documents. We didn’t perform the publication bias of RFS and DMFS because of no more than four studies included in each analysis.

## Discussion

The results of the study illustrated that elevated CDC20 expression indicated unfavorable prognosis OS of various malignancy patients, which was consistent with Wang et al.’s study (32). Our study further found that high CDC20 expression was connected with poor RFS and DMFS in malignancies. What’s more, we did more subgroup analyses stratified by follow-up time, type of cancers, different countries, respectively. Then we discovered that high expressing CDC20 was related to poor OS in lung cancer.

The above conclusions appear to be rational and understandable in line with the current agreement that as a chief cancer promoter, CDC20 can promote the abnormal growth and tumorigenesis of different kinds of tumors, such as lung cancer, which can serve as a promoter for regulating the progression of G1/S transition and the survival of cancer cells (10, 33). What’s more, Zheng et al. had demonstrated that besides the initiation, the presence of CDC20 is essential for tumor maintenance (34), which jointly contributes to the unfavorable prognosis in patients with elevated CDC20 expression level. Furthermore, Gao et al. and Li et al. showed that CDC20 could be useful for the treatment of osteosarcoma and might be a promising solution for the treatment of osteosarcoma with some chemotherapeutics insensitivity (35, 36). Notably, creasing studies found that CDC20 played a critical role in hematological malignancies as a prognostic factor and therapeutic target (37). Thus, CDC20 is likely to act as a prognosis factor for the occurrence, maintenance, drug resistance of malignancy tumors.

Furthermore, we also explored the association between the CDC20 expressing levels with the prognostic value among various cancers. But just on account of the restricted amounts of selected research, we only evaluated the prognostic value of CDC20 in lung cancer. And the result showed that higher CDC20 level implicated an unfavorable OS in lung cancer. Moreover, although we found that there existed sight heterogeneity among non-lung cancer group, it presented significant correlation between the elevated expression levels of CDC20 and the poor OS of non-lung cancer (HR 2.67, 95%CI 2.08-3.44, p <0.001). Based on the above, we believe that the prognostic role of CDC20 in diverse cancers is significant.

With regard to RFS, DMFS, disease free survival (DFS) and progression- free survival (PFS), these are all essential parameters reflecting the procession of malignancy. As all the included studies did not present any data about DFS and PFS, we just made analysis about RFS and DMFS. The outcomes of this meta-analysis revealed that higher CDC20 level implicated a poor RFS and DMFS in tumor patients. What’s more, due to the fact that only two researched were enrolled to appraise the connection among CDC20 expressing levels and RFS, DMFS respectively, more researched are essential to investigate the connection about CDC20 and the development of cancer.

Except for the encouraging results, there are several limitations among this quantitative meta-analysis. First, there was a risk of publication bias, as some studies with small sample sizes or negative results may not have been published. Second, there may be a certain publication bias within some of the included studies, as any negative results are less likely to have been reported. Finally, the results cannot fully represent all solid tumors and hematological malignancies since the types of cancer covered by included trials are incomplete, and further clinical trials are needed to explore.

## Conclusions

In sum up, this meta-analysis suggested that higher expressing levels of CDC20 was correlative to poor prognosis of OS, RFS and DMFS among difference kinds of malignancy patient. In brief, our current study is the most comprehensive meta-analysis that systemically explores the incontrovertible evidence of the prognosis value of CDC20 in various malignancy patients. More related works still need to improve the understanding of CDC20 expression and prognosis in difference cancer types.

## Data availability statement

The raw data supporting the conclusions of this article will be made available by the authors, without undue reservation.

## Author contributions

FX and GX designed this study. FX and XY contributed to the literature search, review, and data extraction. FX and GX conducted the statistical analyses. FX and XY contributed to the manuscript drafting. FX and GX contributed to the manuscript revision. XY offered the funding. All authors accepted the eventual manuscript. All authors read and approved the final manuscript.

## Funding

The meta-analysis was supported by Beijing Medical Award Foundation (YXJL-2020-0972-0424), Natural Science Foundation of Sichuan (No. 2022NSFSC0837) and Science and Technology Project of Chengdu (No. 2022-YF05-01811-SN).

## Conflict of interest

The authors declare that the research was conducted in the absence of any commercial or financial relationships that could be construed as a potential conflict of interest.

## Publisher’s note

All claims expressed in this article are solely those of the authors and do not necessarily represent those of their affiliated organizations, or those of the publisher, the editors and the reviewers. Any product that may be evaluated in this article, or claim that may be made by its manufacturer, is not guaranteed or endorsed by the publisher.
